# Creation of Curved Nanostructures Using Soft-Materials-Derived Lithography

**DOI:** 10.3390/nano10122414

**Published:** 2020-12-03

**Authors:** Hyun-Ik Jang, Hae-Su Yoon, Tae-Ik Lee, Sangmin Lee, Taek-Soo Kim, Jaesool Shim, Jae Hong Park

**Affiliations:** 1 National NanoFab Center, 291 Daehak-ro, Yuseong-gu, Deajeon 34141, Korea; hijang2018@nate.com (H.-I.J.); hsyoon2018@nate.com (H.-S.Y.); 2 Nanoin Inc., 291 Daehak-ro, Yuseong-gu, Deajeon 34141, Korea; 3 Joining R&D Group, KITECH, 156 Gaetbeol-ro, Yeonsu-gu, Incheon 21999, Korea; tilee@kitech.re.kr; 4 Department of Mechanical Engineering, KAIST, 291 Daehak-ro, Yuseong-gu, Deajeon 34141, Korea; lsm417@kaist.ac.kr (S.L.); tskim1@kaist.ac.kr (T.-S.K.); 5 School of Mechanical Engineering, Yeungnam University, 280 Daehak-ro, Gyeongsan-si, Gyeongbuk 38541, Korea

**Keywords:** nanostructure, nanopatterning, soft lithography, soft material, swelling

## Abstract

In this study, curved nanostructures, which are difficult to obtain, were created on an Si substrate through the bonding, swelling, and breaking processes of the polymer and silicone substrate. This method can be utilized to obtain convex nanostructures over large areas. The method is simpler than typical semiconductor processing with photolithography or compared to wet- or vacuum-based dry etching processes. The polymer bonding, swelling (or no swelling), and breaking processes that are performed in this process were theoretically analyzed through a numerical analysis of permeability and modeling. Through this process, we designed a convex nanostructure that can be produced experimentally in an accurate manner.

## 1. Introduction

Nanoscale structures are recognized for their diverse application and versatility in a variety of fields such as electronics and communications technology, optical technology, biotechnology, environment and energy technology, and national defense technology [1,2,3,4]. There has been an increasing interest in nanostructure processing with curved surfaces. This is because nanostructures used in essential parts such as light guide panels, lenses, labs-on-chips, among others required in displays, optics, and nano-biotechnology are decreasing in size but increasing in precision. There is a large demand for highly reproducible nanostructures. Previously, methods such as the lithography thermal reflow technique, gray-tone photolithography, diamond milling, and beam direct writing have been suggested as methods for the creation of curved micro- and nano-scale structures [5,6,7,8,9]. However, there are still some unresolved issues regarding the use of the methods for the fabrication of high-quality curved structures. In addition, because these methods are heavily equipment-dependent and require high processing costs, and taking into account processing time and energy efficiency, they are not adequate for mass-production. Such methods show low structural reproducibility and have a limitation in scaling down to smaller structures. Park et al. reported a method that can implement nano-lenses in an area of 1 μm or less us organic vapor deposition. Although the above method does not require the use of expensive semiconductor processes, the dimensions and shapes of the standardized nano-lenses are not uniform because they are crystallized on a specific surface-treated substrate by controlling organic vapor in a vacuum. Additionally, lenses can be aggregated together [10]. 

There is an increasing interest in soft lithography approaches, which are capable of mass replicating nanostructures [2,4,9,10,11,12,13,14,15,16,17]. Kim et al. demonstrated that line-type nanostructures can be transferred uniformly onto SiO_2_ substrate using the PDMS (polydimethylsiloxane) swelling method/applied soft lithography technique [18]. These technologies semi-permanently conserve the nanostructure’s Si master mold, which requires high processing costs. They are advantageous for mass production as they enable infinitely reversible and repeatable processes. Additionally, since the processing precision of the semiconductor process is maintained, the error range of the fabricated nanostructures is significantly small. With such advantages, several functional types of soft lithography have been developed, including technology for replication between different types of material, 3D replication technology, etc. [14,19].

In this study, convex structures were uniformly formed on a silicon substrate using the bonding, swelling, and breaking process of modified polydimethylsiloxane (MPDMS), which was replicated from an Si master mold. The bonding, swelling (or lack thereof), and breaking processes of the PDMS nanostructures on the silicon substrate were analyzed theoretically with permeability modeling. Through our experimental results and theoretical analysis, we have developed a novel fabrication method for convex nanostructures. Our method allows for easy and accurate fabrication, suggesting the method’s applicability. 

## 2. Materials and Methods 

### 2.1. Fabrication of SPDMS (Soft PDMS)

The soft polydimethylsiloxane (SPDMS) (Dow Corning) mixture of Sylgard 184 elastomer and curing agent was fabricated in a mass ratio of 10:1 and stirred using a glass rod for approximately 5 min in a disposable container. Air bubbles in the mixed SPDMS were removed in a vacuum oven (OV-12, JEIO Tech, Deajeon, Korea) for 30 min, and the SPDMS mixture was poured into a thermally cured hard PDMS (HPDMS) substrate. Subsequently, it was heated in an oven at 70 °C for 1 h. For reference, the Young modulus of SPDMS is about 2 MPa [19]. 

### 2.2. Fabrication of HPDMS (Hard PDMS)

The HPDMS mixture was prepared by mixing VDT-731 (vinylmethylsiloxane-dimethylsiloxne copolymer, trimethylsiloxy terminated, Gelest Inc., Morrisville, PA, USA), a Pt catalyst (platinum1,3-divinyl-1,1,3,3-tetramethyl-disiloxane-complex, Karstedt catalyst in Xylene, JSI Silicon Corp, Seongnam, Republic of Korea), and a monomer (2,4,6,8-tetramethyl-2,4,6,8-tetravinylcycloterasiloxane, Sigma Aldrich Corp, Saint Louis, MO, USA) at a ratio of 3.4 g:2 drops:1 drop. The mixture was placed in a disposable container and mixed for 1 min using a glass rod. Air bubbles in the mixed HPDMS were removed in the vacuum oven at room temperature for approximately 5 min. Next, 1 g of HMS-301 (methylhydosiloxane-dimethylsiloxane copolymer, trimethylsiloxane terminated, Gelest Inc., Morrisville, PA, USA) was mixed into the HPDMS mixture. It was then stirred slowly with a glass rod to prevent air bubbles. The HPDMS mixture was poured into the Si master with nanostructures and cured by heating in an oven at 70 °C for 20 min. The tensile modulus of the HPDMS was 6.32 MPa in air, and significantly decreased to 0.60 MPa when swelled by the THF solvent.

### 2.3. Fabrication and Treatment of MPDMS (Modified PDMS)

**Figure 1:**
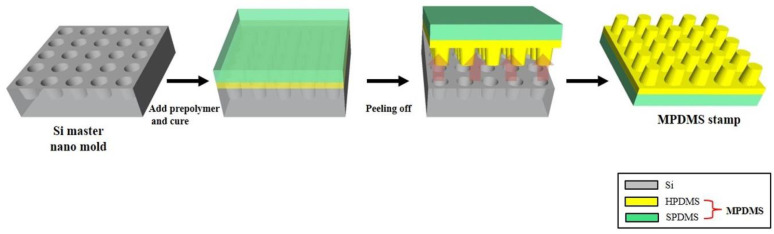
Schematic illustrations of the replica molding process for fabricating the modified PDMS (MPDMS) stamp from the Si master nano-mold.

The modified PDMS (MPDMS) was composed of SPDMS (soft polydimethylsiloxane) on HPDMS (hard polydimethylsiloxane), which is shown in Figure 1. The thermally curable MPDMS stamp was fabricated by the replica molding process (one of the lithography methods) using an Si substrate with a nano-scale structure as the master mold [14]. Subsequently, the Si substrate was cleaned using ultrasonication for 1 min in each of acetone, ethanol, and deionized water, sequentially. The Si substrate was completely dried in N_2_ gas. Next, the prepared MPDMS stamp and Si substrate were treated with plasma on each surface for 2 min 30 s using oxygen at a flow rate of 800 cc per minute and 85 watts of energy using a plasma cleaner (CUTE, FEMTO SIENCE) for the irreversible bonding process. Immediately after the Si substrate and MPDMS surfaces were exposed to the plasma, the MPDMS stamp was carefully placed on the Si substrate. Subsequently, the MPDMS stamp placed on the Si substrate was heated in an oven at 70 °C for 5 min. As a result, the Si substrate and the MPDMS stamp were bonded.

### 2.4. Fabrication of Fully Swelled MPDMS 

The bonded MPDMS stamp and Si substrate were dipped for 5 min in swelling organic solvents such as tetrahydrofuran (THF, swelling ratio: 1.38), hexane (swelling ratio: 1.34), 1,2-dichlorobenzene (1,2-DCB, swelling ratio: 1.28), all of which have high penetration properties for PDMS. The bonded MPDMS stamp and Si substrate were completely dried in N_2_ gas. Subsequently, the MPDMS stamp and the Si substrate were separated from the edge of the bonded plane.

### 2.5. Fabrication of Convex-Type Nanostructures on Si 

The polymer transferred onto the Si substrate was etched using a deep silicon etcher (TCP-9400DFM, LAM) in 17 mTorr (2.2644 Pa) of Cl_2_/HBr/O_2_, with a main power of 300 watts and a bottom bias of −225 watts. Subsequently, the remaining polymer was completely removed in a polymer remover solution (Dynasolve 218, DYNALOY) at 45 °C with an agitation of 150 rpm for 2 h. As a result, convex nanostructures were uniformly formed on the Si substrate.

## 3. Results and Discussion

### 3.1. Curved Nanostructure Creation via the Bonding, Swelling (or Lack Thereof), and Breaking Process from Replica Molding 

In this study, we performed convex nanostructure fabrication using PDMS nanopattern transfer technology. PDMS nanopattern transfer technology is divided into two steps. The first step is the replica molding step [13]. Figure 1 illustrates the fabricating process of replicating MPDMS stamps through molding and curing processes using an Si-based master mold with micro- and nano-scale structures [14]. We defined the nanostructured HPDMS (hard polydimethylsiloxane) on the SPDMS backing substrate as modified PDMS (MPDMS). 

**Figure 2:**
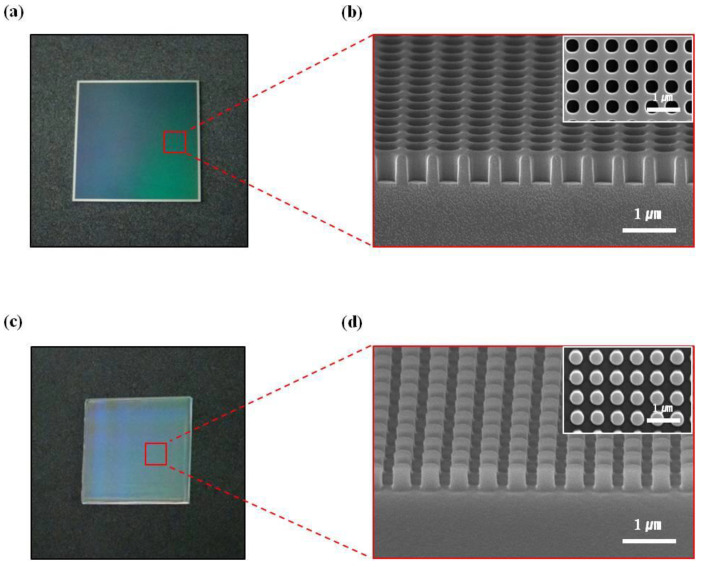
(**a**) Optical image of the uniformly etched Si master nano-mold. (**b**) Cross-sectional and top SEM images of arrayed circular nano-holes with a relief depth of 520 nm. (**c**) Optical image of the replicated MPDMS stamp from the Si master nano-mold. (**d**) Cross-sectional and top SEM images of the replicated MPDMS stamp with a circular pillar structure.

Figure 2a is an optical image of an Si master nano-mold with a 1 inch × 1 inch size. Figure 2b shows the nanostructure of Figure 2a. Nanostructures of circular holes with a diameter of 400 nm, an interval of 200 nm, and a depth of 520 nm were fabricated on an Si wafer using the photolithography and etching process with an inductively coupled plasma reactive ion etcher (ICP-RIE). Figure 2c shows an optical image of the MPDMS stamp, which was replicated from the Si master nano-mold in Figure 2a and whose structure is reversed through the replica molding method. Figure 2d shows the nanostructure of Figure 2c.

**Figure 3:**
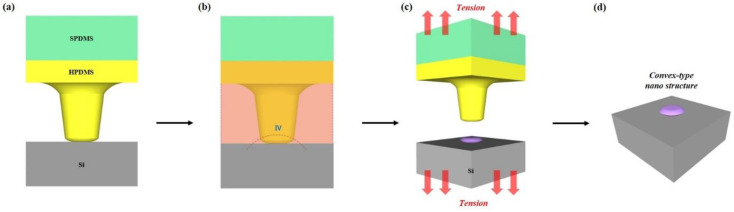
Schematic process flow to fabricate the convex nanostructure from the MPDMS stamp: (**a**) The irreversible bonding step; (**b**) the fully swelling step; (**c**) the breaking step on applied tension; (**d**) the resulting convex nanostructure transferred to the Si substrate.

Figure 3a is a schematic diagram showing the bonded state of the oxygen-plasma-treated MPDMS stamp and Si substrate. Figure 3b is a schematic diagram of a state where the MPDMS stamp is dipped into THF (tetrahydrofuran) and THF has completely penetrated into the entire MPDMS stamp. If enough time is given for THF to penetrate into the MPDMS stamp, the concentration of THF in the MPDMS stamp remains constant regardless of the penetration path and the penetration area. In other words, the degree of breakdown of the bonds between HPDMS’s molecular chains is the same throughout the entire polymer. If a relatively low critical tensile stress is applied in this state, then the probability of a fracture occurring at the bottom of the HPDMS pillar with the smallest cross-sectional area is high [20]. As above, if the MPDMS stamp does not swell at all, the state of the bonds between HPDMS’s molecular chains is the same throughout. If a relatively high critical tensile stress is applied in this state, then the probability of a fracture occurring at the bottom of the HPDMS pillar with the smallest cross-sectional area is high. Figure 3c shows the broken MPDMS structure when critical tensile force is applied to Figure 3b. Figure 3d shows the MPDMS convex nanostructure transferred to a Si substrate.

### 3.2. Curved Nanostructure Creation via the Bonding, Swelling (or Lack Thereof), and Breaking Process from Replica Molding

**Figure 4:**
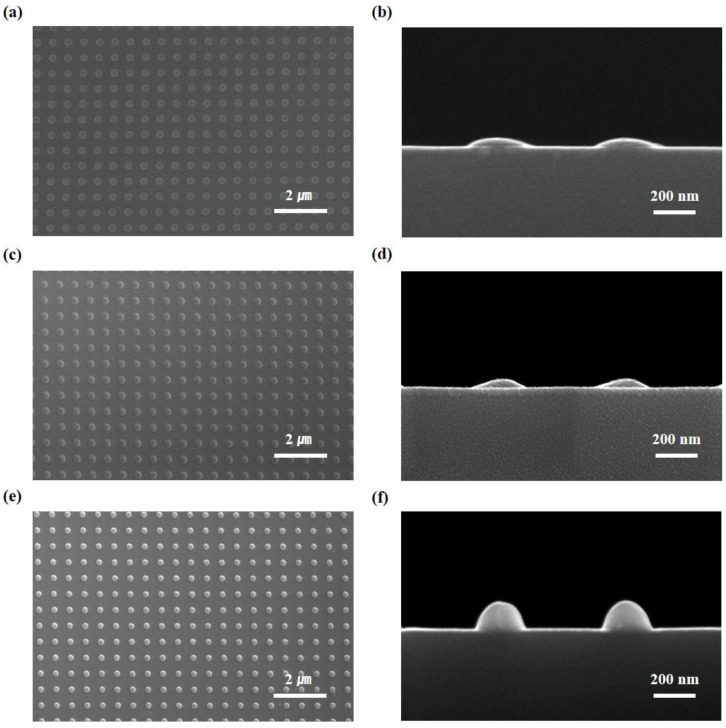
Fabricated convex nanostructures after transfer to the Si substrate by the MPDMS bonding, swelling (i.e., no swelling or full swelling), and breaking process (used as a hard mask against dry etching): (**a**,**b**) are SEM images of the top and cross-sectional views, respectively, of transferred convex patterns (diameter of 300 nm, spacing of 300 nm, and height of 40 nm) onto the Si substrate by the bonding and breaking process (no swelling). (**c**,**d**) are SEM images of the top and cross-sectional views of fully swelled and transferred convex patterns, respectively. (**e**,**f**) present convex Si nanostructures formed by deep reactive ion etching (RIE) through the MPDMS hard masks with convex patterns as shown in (**c**,**d**).

Figure 4a,b shows the top and cross-sectional views, respectively, of convex patterns of MPDMS stamps (diameter of 300 nm, spacing of 300 nm, and height of 40 nm) uniformly transferred onto the Si substrate through the bonding, no swelling (the swelling was skipped) and breaking process. Figure 4c,d shows the top and cross-sectional views, respectively, of the convex patterns of the MPDMS stamp uniformly transferred to the Si substrate through the bonding, full swelling, and breaking process. As the transferred MPDMS convex nanopatterns are used as hard masks for the etching process, convex Si nanostructures (diameter of 250 nm, spacing of 350 nm, and height of 160 nm) that are difficult to form by conventional photo lithography were easily formed. An important parameter that affects the change of curvature is determined by the degree of swelling. Swelling is determined by the concentration of THF, the time of swelling, and the shape and size of the nanostructure. For example, in the case of full swelling, in which HPDMS nanostructures are completely penetrated by THF, the effect is the same as no swelling. However, in the case of the full swelling step, less energy is required for the breaking step than in the case of no swelling step (i.e., swelling is skipped). Therefore, the uniformity and accuracy of the nanostructure formation step are improved even with a small force. Figure 4e,f shows the Si nano lens structures, which were formed after deep RIE using the convex patterns in Figure 4c,d as hard masks for the etching process. The convex nanostructures fabricated through this process are used as a functional structure in biological and medical fields as well as in the development of optical lenses and sensors [21]. In this study, experiments were conducted with one type of nano-structure. However, by using the bonding, full swelling, and breaking process, curvature is not controllable, but is determined naturally via swelling and breaking mechanics. Since the diameter and height of the fabricated curved nano-structure depends on the diameter of the initial nano-structure of the Si master nano-mold, it is thought that if a smaller Si nano-mold structure is used, a smaller-diameter curved structure can be fabricated.

### 3.3. Mechanical Modulus Measurement of the HPDMS 

A dynamic mechanical analyzer (DMA) (DMA 8000, Perkin Elmer, Waltham, MA, USA) was used to measure the tensile modulus of the HPDMS. In order to investigate the effect of THF swelling on the mechanical properties of HPDMS, a ceramic-coated fluid bath of the DMA machine was utilized. Among DMA testing configurations, the film tension mode was adopted because the tensile elastic response is important when analyzing stress concentration in the nanostructures during the initial stage of the transfer process. The HPDMS specimens were fabricated with dimensions of 20 mm length, 5 nm width, and 1mm thickness using an aluminum mold. The HPDMS solution was thoroughly mixed and then poured into rectangular grooves. After degassing trapped air in the mixed solution under vacuum, thermal curing was conducted at 90 °C for 30 min. With the gauge length of 10 mm, the dynamic actuation was conducted both in the air and in the THF solvent. For consistency, the measurement in the THF solvent started after 2 min of immersion. The measured tensile modulus values were averaged with results from at least five specimens.

**Figure 5:**
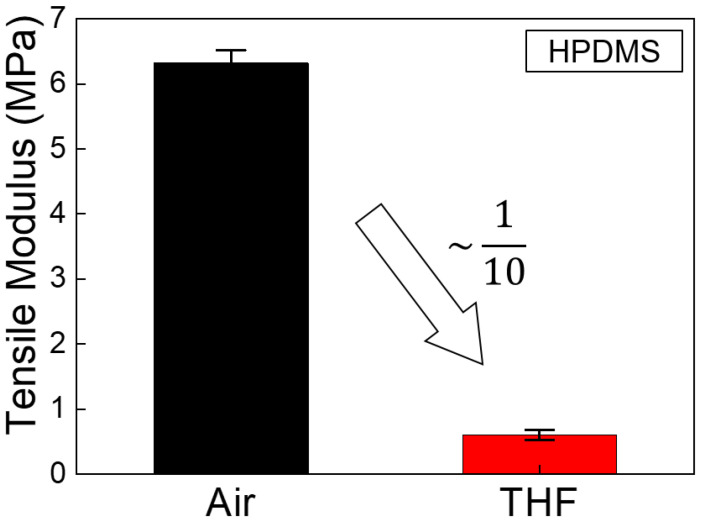
Tensile modulus of HPDMS in air and in THF.

Figure 5 shows the modulus measurement result of the HPDMS specimens under ambient conditions and under the THF-swelled condition. The tensile modulus of the HPDMS was 6.32 MPa in the air and significantly decreased to 0.60 MPa when swelled by the THF solvent. It is verified that the THF treatment greatly weakens the intermolecular attraction among the polymeric chains. Note that the modulus value during 3 min of the cyclic measurement was kept constant, meaning that the mechanical stiffness was saturated to the modulus level of 0.60 MPa.

### 3.4. Numerial Analysis of Permeability and Modeling in the Bonding, Controlled Swelling, and Breaking Process

A stationary stress analysis was conducted to validate the failure process of convex nanostructures. Interpenetrating swelling properties of the HPDMS and polymer networks were obtained from the experiment (Figure 5) and were utilized to carry out a numerical simulation by using Young’s modulus (tensile modulus) of the fully swelled HPDMS. We assume that Young’s modulus is the tensile modulus in an environment where tensile force is applied. The failure process was studied by examining two main tensors: diffusion of the chemical solvent and stationary stress under the specific swelled conditions.

#### 3.4.1. Concentration Diffusion Analysis

The concentration equation is defined as:

(1) $$\frac{\partial C}{\partial t}+\vec{U}\cdot \nabla C=\nabla \cdot \left(D\nabla C\right)+R$$

where *C* is the concentration (mole/m^3^) and D is the diffusion coefficient (m^2^/s). In addition, $\vec{U}$ is the fluid velocity and *R* is the net rate at which *C* is produced in a chemical reaction. In this simulation, convection is suppressed to obtain a pure diffusion profile with time. The reaction of the PDMS polymer does not take place during swelling.

#### 3.4.2. Stationary Stress Analysis

The stationary stress–strain equation is expressed as:

(2) $$\nabla \cdot \sigma +F_{v}=0$$

(3) $$\sigma =\sigma_{0}+C:\left(\varepsilon -\varepsilon_{0}-\alpha \theta \right)$$

(4) $$\varepsilon =\frac{1}{2}\left(\nabla u+{\left(\nabla u\right)}^{T}\right)$$

where σ is the stress tensor, $F_{v}$ is the body force, ε is the strain tensor, $\sigma_{0}$ and $\varepsilon_{0}$ are the initial stress and strain tensors, respectively, α is the thermal expansion, $\theta =T-T_{ref}$, u is the displacement field, and C is the 4th-order elasticity tensor.

This displacement field gradient can be defined as:

(5) $$\nabla u=\left[\begin{array}{l} \frac{\partial u}{\partial X}\;\frac{\partial u}{\partial Y}\;\frac{\partial u}{\partial Z}\\ \frac{\partial v}{\partial X}\;\frac{\partial v}{\partial Y}\;\frac{\partial v}{\partial Z}\\ \frac{\partial w}{\partial X}\;\frac{\partial w}{\partial Y}\;\frac{\partial w}{\partial Z} \end{array}\right]$$

where (*X, Y, Z*) are material coordinates and (x, y, z) are spatial coordinates.

#### 3.4.3. Numerical Simulation and Modeling of Permeability

In this study, two failure modes, such as fracture at upper regions and fracture at lower regions of pillar type nanostructures, were investigated by using experimental data as numerical input data. We studied how to propagate the concentration of chemical solvent with time before the stationary stress analysis was carried out to study the failure process.

**Figure 6:**
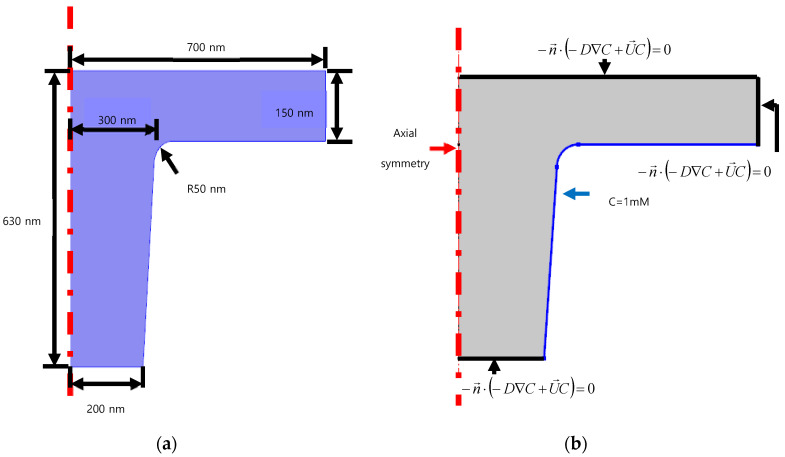
Schematic of cylindrical bead nanostructure: (**a**) geometry; (**b**) boundary conditions of concentration simulation.

We used the diffusion coefficient 1E-16 m^2^/s for the HPDMS and chemical solvents [21]. Figure 6 shows the schematic of a cylindrical bead nanostructure. For the boundary conditions of concentration diffusion, the axial symmetry condition is used at the axial line, whereas the bottom regions and the top-right are set as the insulation boundary. Only the constant concentration is provided at the blue regions as *C* = 12.3 M.

**Figure 7:**
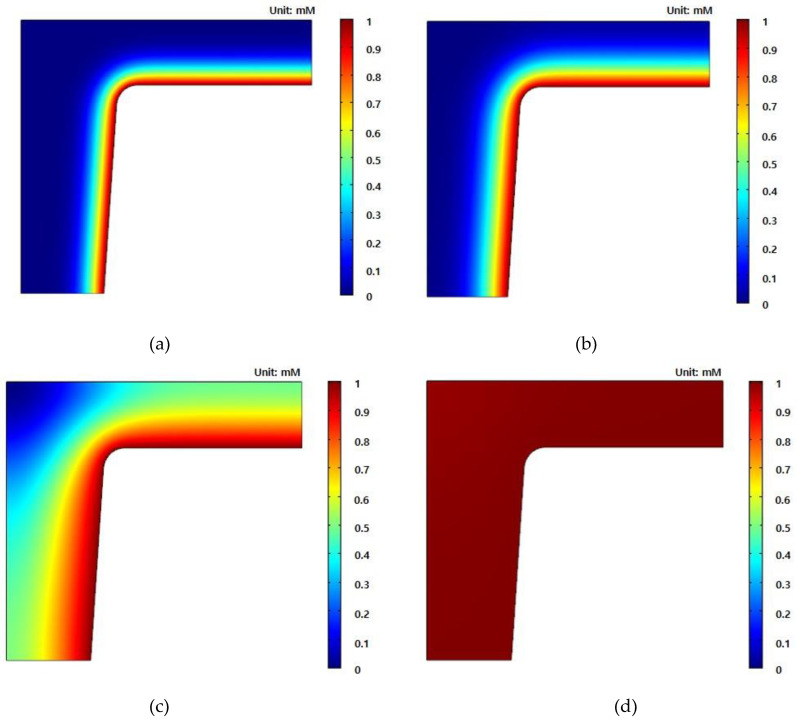
Concentration profile of a cylindrical bead nanostructure with time: (**a**) 1 s; (**b**) 5 s; (**c**) 10 s; (**d**) 120 s.

The concentration propagation with time is revealed in Figure 7. The PDMS swelling due to chemical solvents starts from the outside before moving into the PDMS (as shown in Figure 7a,b) and the concentration still does not reach the top of the center of the nanostructure in Figure 7c. Finally, the PDMS is fully swelled in Figure 7d. We also conducted a stationary stress analysis by using the numerical results of concentration diffusion. The result at 10 s (Figure 7c) was used as the partially swelled condition and 120 s (Figure 7d) was used as the fully swelled condition.

**Figure 8:**
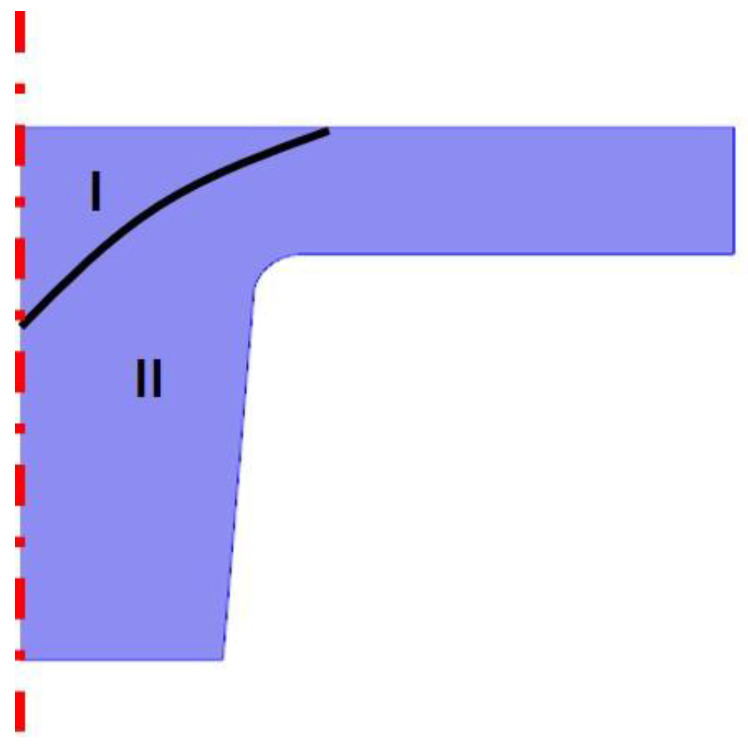
The schematic of the partially swelled nanostructure.

In Figure 8, the partially swelled regions are divided into region I (non-swelled region) and region II (fully swelled region). The classified curve was obtained from the Bezier polygon, which is based on {r, z} = {[−1E−7m, 2E−7m], [1E−7, 6E−7], [5E−7, 8E−7]}. For boundary conditions, a 2D axial symmetric boundary was applied to the axisymmetric line. A fixed boundary condition was applied to the bottom of the pillar nanostructure and the prescribed displacement of 80 nm for the partially swelled condition and 170 nm were set to the top of nanostructure. All other boundaries were used as free boundary conditions. In Figure 8, the schematic of the partially swelled nanostructure is shown for simulation of the swelling condition. Area I of the HPDMS is dried, as E = 6.32 MPa, and area II of the HPDMS is swollen, as E = 0.6. The Poisson ratios are v = 0.45 and 0.47 for the dried condition and the swollen condition, respectively.

**Figure 9:**
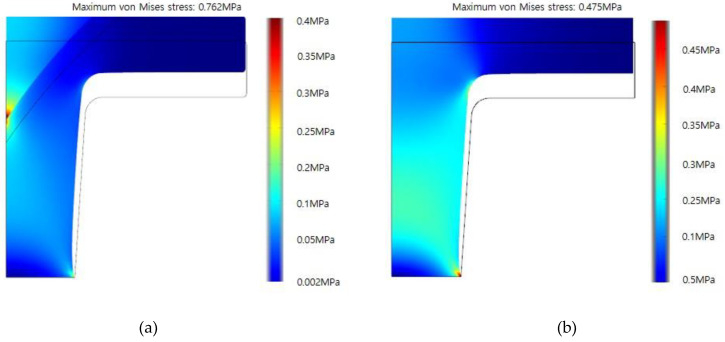
Stress analysis results for (**a**) the partially swelled condition and (**b**) the fully swelled condition.

Figure 9 shows the simulation results for the partially swelled PDMS (Figure 9a) and for the fully swelled PDMS (Figure 9b). The maximum stress of the partially swelled PDMS is 0.762 MPa at the edge of the bottom, whereas the maximum stress of the fully swelled PDMS is 0.475 MPa. It was reported that the maximum strengths of dry PDMS and swollen PDMS are 0.76 and 0.45 MPa, respectively [22]. As compared to the literature, the maximum stresses for both the partially swelled PDMS and the fully swelled PDMS are greater than the material strength. This means that the partially swelled PDMS breaks near the top of the nanostructure and the fully swelled HPDMS fractures at the bottom of the nanostructure.

## 4. Conclusions

In this study, convex nanostructures over large areas were formed on a silicon substrate by using the bonding, swelling, and breaking of MPDMS that was replicated from an Si master mold. In addition, the bonding, swelling (or lack thereof), and breaking process of the PDMS nano-mold on the silicon substrate was analyzed theoretically through numerical analysis of permeability and modeling. Through this process, we have developed a novel fabrication method for convex nanostructures that can easily and accurately be fabricated, which usually represents a difficult engineering problem. Easily fabricated, curved nanostructures can be essential parts required in the fields of displays, optics, and nano-bio diagnosis, and they can play an important role in light guide panels, lenses, and labs-on-chips.
